# A pooled analysis of host factors that affect nucleotide excision repair in humans

**DOI:** 10.1093/mutage/geae028

**Published:** 2024-12-13

**Authors:** Congying Zheng, Sergey Shaposhnikov, Andrew Collins, Gunnar Brunborg, Amaya Azqueta, Sabine A S Langie, Maria Dusinska, Jana Slyskova, Pavel Vodicka, Frederik-Jan van Schooten, Stefano Bonassi, Mirta Milic, Irene Orlow, Roger Godschalk

**Affiliations:** Department of Pharmacology and Toxicology, NUTRIM School of Nutrition and Translational Research in Metabolism, Maastricht University, 6200 Maastricht, The Netherlands; Norgenotech AS, Ullernchassern, 64/66, 0379 Oslo, Norway; Oslo Cancer Cluster, Ullernchausseen, 64/66, 0379 Oslo, Norway; Norgenotech AS, Ullernchassern, 64/66, 0379 Oslo, Norway; Oslo Cancer Cluster, Ullernchausseen, 64/66, 0379 Oslo, Norway; Norgenotech AS, Ullernchassern, 64/66, 0379 Oslo, Norway; Norgenotech AS, Ullernchassern, 64/66, 0379 Oslo, Norway; Department of Pharmaceutical Sciences, School of Pharmacy and Nutrition, University of Navarra, 31008 Pamplona, Spain; Department of Pharmacology and Toxicology, NUTRIM School of Nutrition and Translational Research in Metabolism, Maastricht University, 6200 Maastricht, The Netherlands; Department of Environmental Chemistry and Health, Health Effects Laboratory, The Climate and Environmental Research Institute NILU, 2027 Kjeller, Norway; Department of the Molecular Biology of Cancer, Institute of Experimental Medicine of the Czech Academy of Sciences, Videnska 1083, 142 00 Prague, Czech Republic; Department of the Molecular Biology of Cancer, Institute of Experimental Medicine of the Czech Academy of Sciences, Videnska 1083, 142 00 Prague, Czech Republic; Biomedical Center, Faculty of Medicine in Pilsen, Charles University, Alej Svobody 1655/77, 32300 Pilsen, Czech Republic; Department of Pharmacology and Toxicology, NUTRIM School of Nutrition and Translational Research in Metabolism, Maastricht University, 6200 Maastricht, The Netherlands; Unit of Clinical and Molecular Epidemiology, IRCCS San Raffaele Roma, 00163 Rome, Italy; Department of Human Sciences and Quality of Life Promotion, San Raffaele University, Unit of Clinical and Molecular Epidemiology, IRCCS San Raffaele Roma, Via di Val Cannuta, 247, 00166, Rome, Italy; Division of Toxicology, Institute for Medical Research and Occupational Health, Ksaverska cesta 2, 10000, Zagreb, Croatia; Memorial Sloan-Kettering Cancer, Department of Epidemiology and Biostatistics, New York, NY 10065, USA; Department of Pharmacology and Toxicology, NUTRIM School of Nutrition and Translational Research in Metabolism, Maastricht University, 6200 Maastricht, The Netherlands

**Keywords:** DNA repair, comet assay, nucleotide excision repair, biomarkers, sex, age, BMI

## Abstract

Nucleotide excision repair (NER) is crucial for repairing bulky lesions and crosslinks in DNA caused by exogenous and endogenous genotoxins. The number of studies that have considered DNA repair as a biomarker is limited, and therefore one of the primary objectives of the European COST Action hCOMET (CA15132) was to assemble and analyse a pooled database of studies with data on NER activity. The database comprised 738 individuals, gathered from 5 laboratories that ran population studies using the comet-based *in vitro* DNA repair assay. NER activity data in peripheral blood mononuclear cells were normalized and correlated with various host-related factors, including sex, age, body mass index (BMI), and smoking habits. This multifaceted analysis uncovered significantly higher NER activity in female participants compared to males (1.08 ± 0.74 *vs*. 0.92 ± 0.71; *P* = .002). Higher NER activity was seen in older subjects (>30 years), and the effect of age was most pronounced in the oldest females, particularly those over 70 years (*P* = .001). Females with a normal BMI (<25 kg/m^2^) exhibited the highest levels of NER, whereas the lowest NER was observed in overweight males (BMI ≥ 25 kg/m^2^). No independent effect of smoking was found. After stratification by sex and BMI, higher NER was observed in smoking males (*P* = .017). The biological implication of higher or lower repair capacity remains unclear; the inclusion of DNA repair as a biomarker in molecular epidemiological trials should elucidate the link between health and disease status.

## Introduction

Nucleotide excision repair (NER) safeguards genomic integrity by the removal of DNA lesions that impede the progress of DNA and RNA polymerases, or induce the formation of disruptive DNA structures [1,2]. Failure to repair these DNA lesions may lead to mutations, and inter-individual differences in NER activity may thus affect disease risk [3,4]. Therefore, a more comprehensive understanding of the factors contributing to inter-individual variation in NER activity is imperative. Indeed, various host-related characteristics, including sex, age, BMI, and smoking, are believed to affect NER activity [4].

Here, we investigated the impact of these host factors on NER in peripheral blood mononuclear cells (PBMCs), which can be obtained with minimally invasive techniques and are well-suited for molecular epidemiological studies. Various endogenous and exogenous factors are relevant to this analysis. The link between sex and NER activity has been the subject of investigation in various studies. Sex-specific differences in NER may contribute to disparities in disease susceptibility and treatment responses [5–7]. Emerging evidence indicates that females display higher NER activity than males [8]. Earlier, Slyskova *et al.* [4] showed that sex and individual antioxidant levels are associated with DNA repair capacity; both BER (base excision repair) and NER were lower in women and increased with high plasma levels of ascorbic acid and carotene. Furthermore, the sex hormone oestrogen was found to modulate sex disparities in cancer prevention and treatment [9,10]. Thus, hormonal influences may underlie these sex-specific distinctions.

Ageing is characterized by the accumulation of cellular damage and a relationship between age and NER activity has been reported [11,12]. For instance, the XPA gene (xeroderma pigmentosum gene, Complementation Group A, chromosome 9, part of NER factor 1 complex), a crucial gene responsible for recognizing DNA lesions, shows reduced expression in aged skin cells. This leads to decreased efficiency in removing cyclobutane pyrimidine dimers (CPDs) induced by UV radiation, underscoring the vital role of NER in preserving genomic stability and prompting further inquiry into the molecular bases of potential age-associated NER decline [13]. Although tissue-specific accelerated ageing is potentially linked to NER-related genes [14], the relationship between human age and NER is not necessarily the same in all cell types and the effect on NER in PBMCs needs further attention.

BMI has recently received attention as a factor influencing NER [4,15,16]. Obesity, characterized by an elevated BMI, is closely linked to chronic inflammation and oxidative stress, both of which could exacerbate DNA damage and thus trigger DNA repair mechanisms. Studies of the interplay between BMI and NER indicate compromised NER capacity in obese individuals [4,16,17]. Also, a deficiency in ERCC1-XPF, a key component of the NER pathway, can lead to lipodystrophy and contribute to metabolic complications [18]. Reduced NER activity in obesity heightens the risk of DNA lesion accumulation, increasing susceptibility to obesity-related diseases, including specific cancers [19].

Finally, smoking is a well-established source of exposure to genotoxic compounds. The carcinogenic compounds in tobacco smoke can induce bulky adducts in DNA. Cigarette smoke exposure reduced NER activity and decreased NER gene expression in human lung cells *in vitro* [20]. Epidemiological studies also demonstrated that reduced NER gene expression increased the risk of lung cancer and head and neck cancer [21–23]. However, the effect of smoking on DNA repair in easily available PBMCs needs further attention.

A better understanding of the combined effects of age, sex, BMI, and smoking on NER activity is important for improving study design of future molecular epidemiological studies and for elucidating factors that determine disease susceptibilities. Therefore, we used the hCOMET database to search for associations between these host factors and measured NER activity in PBMCs.

## Materials and methods

### Database

The data were obtained from the hCOMET database [24], which contains DNA repair data from six molecular epidemiological studies [4,25–29]. Additional unpublished data provided by these authors were included; all these studies employed the comet-based *in vitro* DNA repair assay for the incision step of NER activity in extracts of PBMCs. One study provided data in which DNA repair was studied by the so-called challenge assay (i.e. induction of damage by benzo(a)pyrene diol epoxide, with the removal of damage monitored by the comet assay over time) [28]. The principles of the *in vitro* and cellular repair assays are different, which may lead to results that need to be differently interpreted [30]. Furthermore, for this study, no additional data (sex, BMI, age, etc.) were available in the database. Two studies without host-factor data were excluded from further analyses. Each study received approval from the local ethics committee for collecting and analysing individual coded data, and all General Data Protection Regulations were respected.

### Assessment of NER by in vitro comet assay

NER was measured by the *in vitro* repair comet assay; the protocols were similar for all labs [31], with one major difference: in five studies the DNA substrate nucleoids contained CPDs (induced by UVC-light) and in one study the substrate nucleoids contained BPDE-DNA adducts (induced by benzo[a]pyrene-diol-epoxide, BPDE). Despite this difference in DNA substrate, there was no difference in the inter-individual variation (SD/average × 100%) for both types of assay (see Table 1) and therefore both types of data were included into the final dataset. Substrate nucleoids were incubated with extracts of PBMCs; DNA repair enzymes in the extracts induced incisions at the sites of substrate DNA lesions, which were subsequently measured with the alkaline comet assay. Standardization within each laboratory was achieved by using a fixed number of cells when preparing extract from isolated PBMCs, or by standardizing the protein concentration of the extract.

**Table 1.** Summary of results in individual laboratories contributing data to the pooled analysis.

A								
Descriptor			Average ± SD (range) or N (%)					
Age (years)			*45.4 ± 15.5*					
Sex (male/female/unknown)			*375/320/8 (53.3/45.5/1.1 %)*					
BMI (kg/m^2^)			*25.0 ± 3.8*					
Smoking habits			*51/284 (15.2/84.8%)*					

B								
Laboratory ID	Agents used to study NER activity	*N* ^a^	Age average ± SD	Sex M/F^b^	BMI kg/m^2^	Smoking habits (Y/N)	Coefficient of variation (%)	Type of scoring
1	UVC	330	46.2 ± 15.1	170/153	25.0 ± 3.8	NR	66%	Visual scoring
2	UVC	34	NR	34/0	NR	NR	89%	Visual scoring
3	UVC	303	25.0 ± 3.8	163/139	46.3 ± 15.1	51/248	87%	% Tail DNA
4	BPDE	36	29.9 ± 10.0	8/28	NR	0/36	87%	% Tail DNA

NR, not reported.

^a^Note that the data provided to the hCOMET database included unpublished data. Therefore, the number of data points in the current analysis may differ from the originally published data.

^b^The sum of females and males may not add up to the total number of data points in the study, because of missing values.

### Statistical analysis

Descriptive statistics of demographic and biological data were reported as frequency with percentage for categorical variables, and as mean with standard deviation for continuous variables. To take into consideration the interlaboratory heterogeneity of NER estimates, all data were normalized towards the individual study means. More detail on this approach can be found in the work previously published on BER [32]. Specifically, for each study, the relative NER values were calculated from individual values of NER divided by the mean NER value of that particular study, that is:


$$\begin{array}{l} Relative\;NER=\frac{Individual\;NER\;value\;in\;study\;X}{Mean\;of\;NER\;values\;in\;study\;X}. \end{array}$$

As a result, all studies were standardized to have a mean value of 1, without affecting each study’s variation. Age was categorized into three arbitrary strata: ≤30 years; 31–60 years; and >60 years. In some subsequent analyses, the category ‘>60 years’ was further divided into 61–70 years and >70 years, because DNA repair in the group of >70 years was found by visual inspection of the data to be different from the rest. BMI was stratified as underweight (BMI = <18.5 kg/m^2^), normal weight (BMI = 18.5–25 kg/m^2^), overweight (BMI = 25–29.9 kg/m^2^), or obese (BMI > 30 kg/m^2^). Differences between groups in the mean level of relative NER were tested using ANOVA with Dunnet’s post hoc test (the homogeneity of variances between groups was confirmed by using the Levene’s test). Considering the explorative aim of this analysis, no correction for multiple comparisons was applied. Differences with a *P* value of < .05 were considered statistically significant. All statistical analyses were conducted using IBM SPSS version 23 and GraphPad Prism 9.

## Results

### Demographics of the study population

Initially, NER activity data were provided for 940 subjects. However, information about all host factors (age, sex, BMI, and smoking habits) was missing for 237 individuals. Therefore, these data points were excluded from the analysis. As a result, data of 703 subjects from the hCOMET database were included in this pooled analysis, comprising 375 males (51 %) and 320 females (49 %) (Information about sex was missing for 8 individuals, which was 1.1% of the final study population). The average age of the study population was 45.4 years, ranging from 18 to a maximum of 80 years (46 subjects had missing age data, accounting for 6.5 % of the final study population). There was no difference in age between males and females (*P* = .09) (Table 1).

### The effect of sex and age on NER activity

**Fig. 1.**
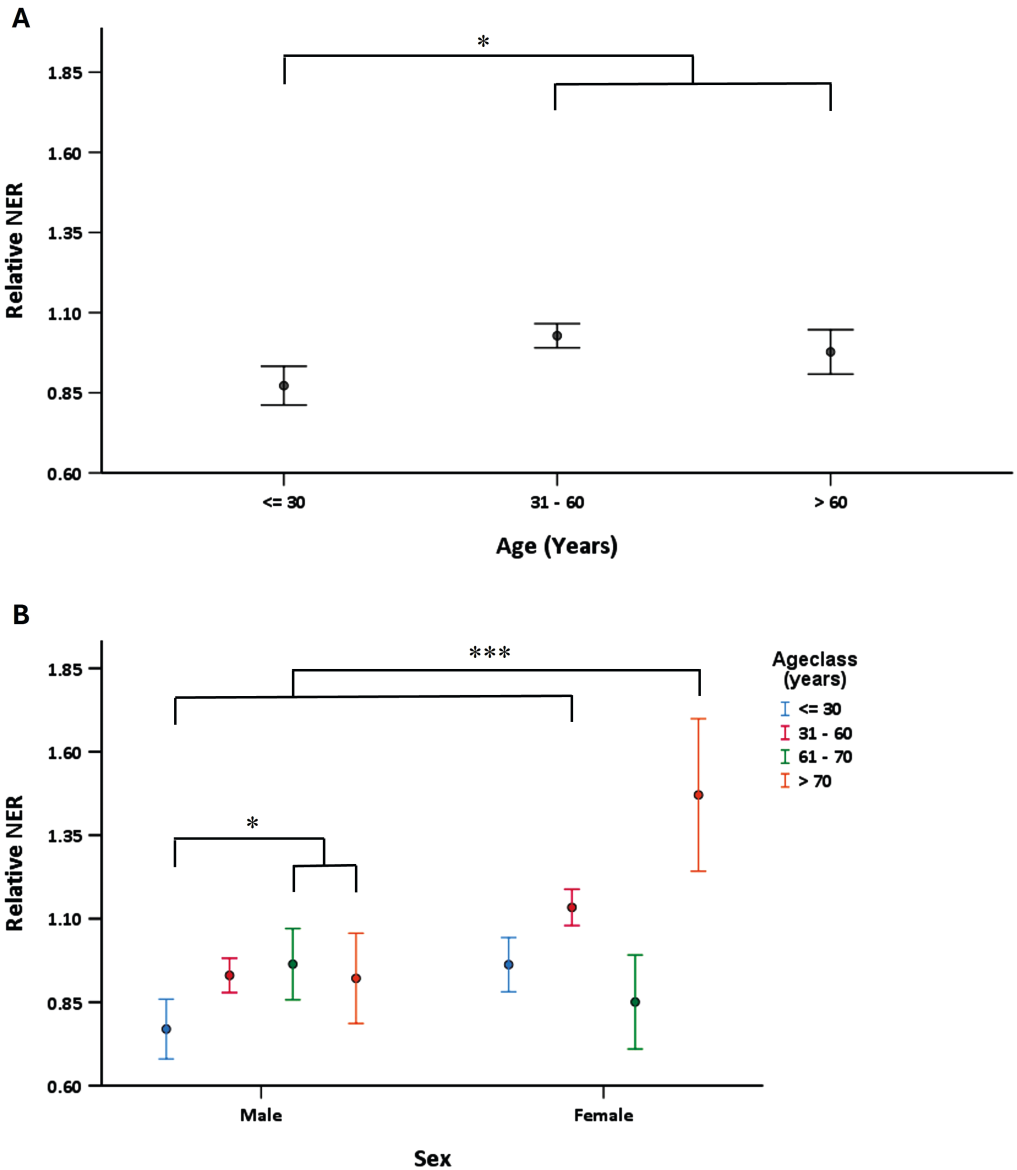
Relationship between various sex/age groups and relative NER. (A) Males and females combined, (≤30 years, 31–60 years, >60 years; *N* = 124, 400, 129, respectively): *P* = .002. (B) Relative NER of males and females stratified by age, ≤30 years, 31–60 years, 61–70 years, >70 years. *N* = 26, 106, 22, 22 respectively (males), *N* = 33, 104, 17, 7 respectively (females) (>60 years vs. ≤30 years, *P* = .04; oldest females (> 70 years) vs. all other sex/age groups, *P* = .001).

The relative NER was higher in females than in males (1.08 ± 0.74 vs. 0.92 ± 0.71; *P* = .002). Because of this sex effect on NER activity, the effects of other variables (age, BMI, and smoking) were subsequently analysed separately for males and females. There was an overall increase in NER with age; subjects older than 30 had a significantly higher DNA repair capacity (*P* = .04) (Fig. 1A). In the males, NER was significantly higher at >60 years compared with ≤30 years (*P* = .04). The oldest females (>70 years, *N* = 7) displayed significantly higher NER compared to all other sex/age groups (*P* = .001) (Fig. 1B).

### NER activity and body mass index

**Fig. 2.**
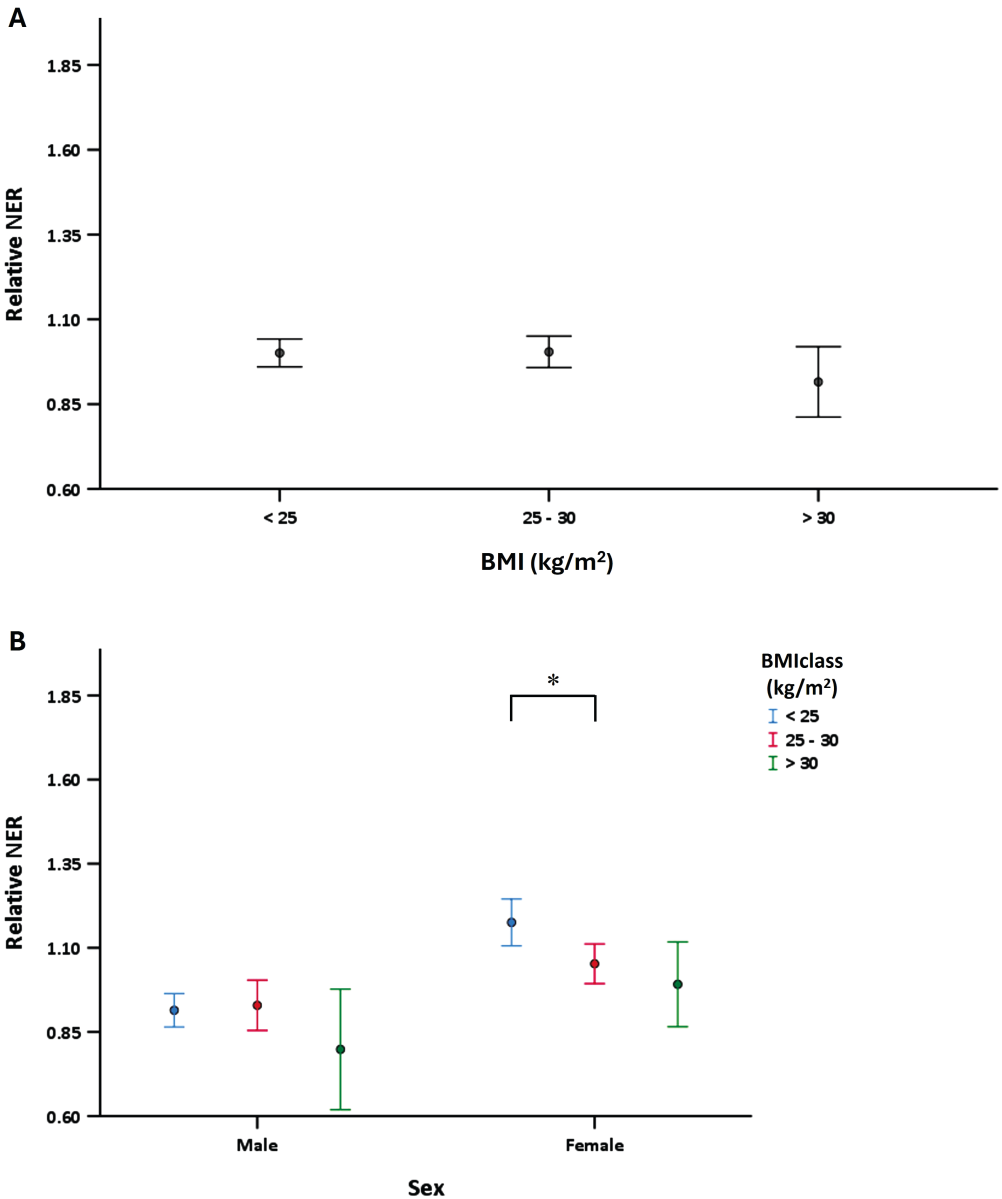
(A) Relationship between BMI and relative NER activity. *N* = 352, 268, and 60 for increasing BMI ranges, no significant effect of NER between different BMI classes (*P* = .61). (B) Relative NER activity is stratified by sex and BMI. *N* = 214, 98, and 22 respectively (males), *N* = 106, 152, and 34 respectively (females), in females, 25 kg/m^2^ vs. 18–25 kg/m^2^, *P* = .033).

A direct relation has not been found between overall BMI and NER (*P* = .61) (Fig. 2A). However, by analysing male and female subjects separately, NER was lowest in females with BMI > 25 kg/m^2^ when compared to females with a normal BMI (18.5-25 kg/m^2^) (*P* = .033) (Fig. 2B). No clear effect of BMI on NER activity was found in men. The highest BMI group showed lower NER activity, but this did not reach statistical significance.

### Smoking habits and NER activity

**Fig. 3.**
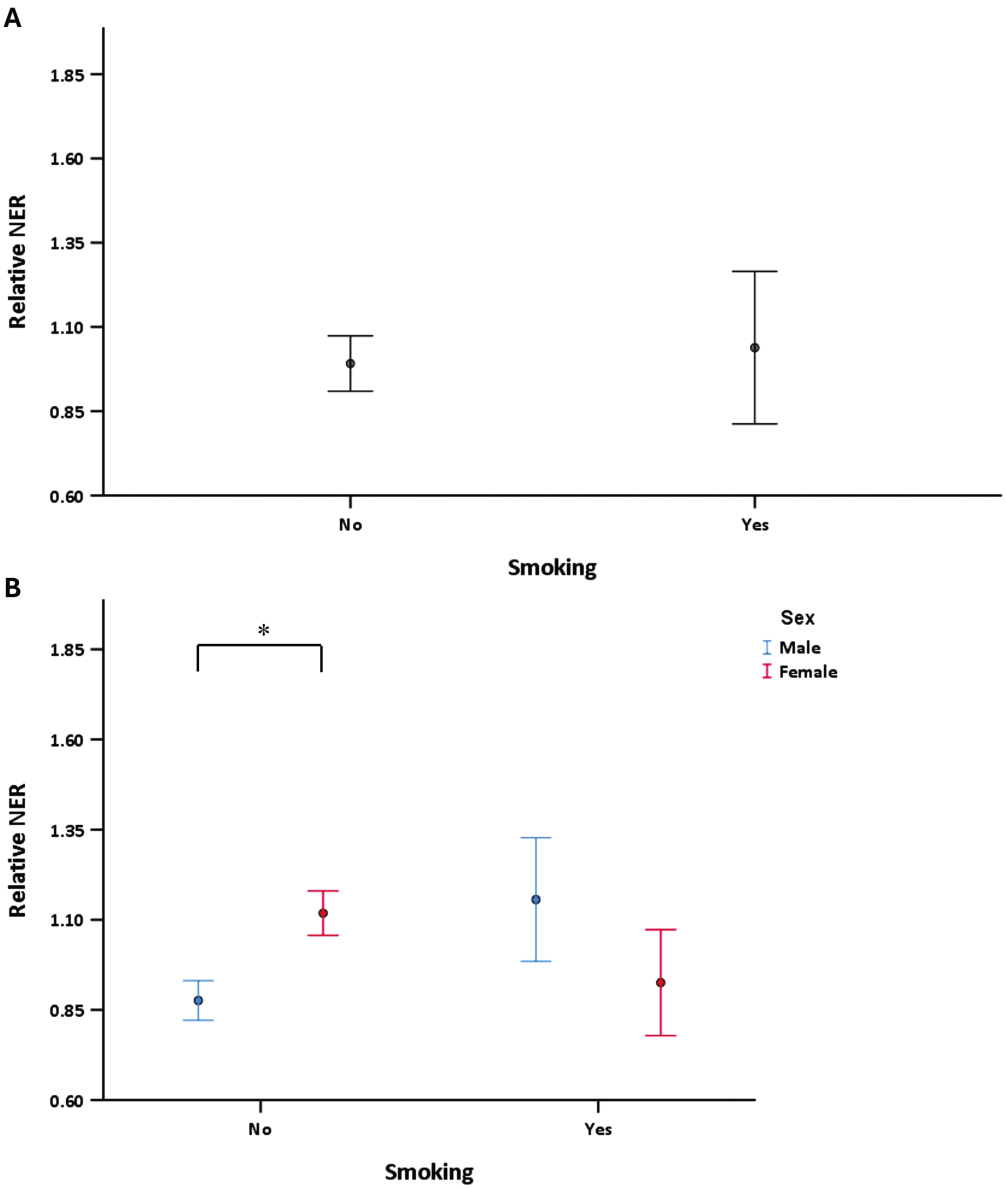
(A) The overall difference between non-smoking (*N* = 312) and smoking (*N* = 59) in relative NER activity (*P* = .106). (B) The relative NER activity of non-smokers and smokers is stratified by males and females (number of subjects: 139, 145, 25, and 26, respectively) (non-smoking, males vs., females, *P* = .017).

No overall difference in NER was observed when comparing subjects who reported being smokers at the time of sampling and non-smokers (*P* = .106) (Fig. 3A). However, NER was higher in male smokers compared to male non-smokers (*P* = .017) (Fig. 3B). An effect of smoking behaviour was not found in females.

## Discussion

DNA repair plays an important role in maintaining genetic stability, because repair is generally considered to lower the amount of DNA damage and subsequent mutagenesis. Indeed, several prospective studies showed that enhanced levels of DNA damage are related to cancer and mortality risks [33–35]. These types of DNA damage can be removed or prevented by either BER or NER. Opattova *et al.* [32] recently showed that BER activity varied between individuals according to various host factors. Here, we applied a similar approach but focussed on how NER is affected by sex, age, BMI, and exposure to cigarette smoke. More insights into how host factors affect NER activity can increase its application as an endpoint in molecular epidemiological studies and can help in the interpretation of results.

The original hCOMET database (established in Cost Action CA15132) actually included six studies that specifically analysed NER activity, and we have used these data to explore the relationship between DNA repair and various host parameters, to understand potential sources of inter-individual variation in NER activities. Three types of modified comet assays were used to measure repair: (1) comet assay-based *in vitro* DNA repair assay with UV damage in the substrate cells, (2) comet assay-based *in vitro* DNA repair assay with BPDE-DNA adducts in the substrate cells, and (3) challenge assay with BPDE. Our previous study on BER, highlighted the differences between the endpoints of these modified comet assays [30]. Specifically, the comet assay-based *in vitro* DNA repair assay measures DNA damage recognition and subsequent incision by DNA repair enzymes, whereas the challenge repair assay additionally reflects the inducibility of DNA repair. In the challenge repair assay, cells are exposed to a DNA-damaging agent to study the removal of damage over time. This exposure by itself may already induce NER activity. Therefore, we excluded the data obtained by the challenge repair assay. One study that used the *in vitro* DNA repair assay with UV damage in the substrate cells did not include data on any of the host factors and was also excluded. As a result, we focussed on the four studies that assessed NER by the comet assay-based *in vitro* DNA repair assay, also providing data on host factors—a total of 703 subjects.

We found notable sex differences in NER activity levels, with women having relatively higher NER activity than men. This difference was particularly pronounced among the older age groups and at low BMI. These findings in PBMCs match some of the known sex disparities in cancer incidence and mortality, which—for various cancer types—are higher in men than in women. For example, the male:female ratio of colorectal cancer varies between 1.2:1 and 1.7:1, and the male/female case incidence ratio for kidney cancer was found to be 2:1 around the world [36–39]. A recent publication reviews the potential mechanism underlying sex disparities in disease risks, including cancer [40]. Overall, these sex differences seem to be determined by genetic, epigenetic, and hormonal factors that mediate cell cycle regulation, oncogenesis, and metastasis. DNA repair protects against mutagenesis and so the differences in NER capacity are likely to contribute to the sex bias in cancer incidence. At the molecular level, both clinical and *in vitro* models exploring the mechanisms of sex disparities have provided evidence that tumour suppressor protein p53 may function differently in males and females, and females are more resistant to the loss of function of p53 [41]. p53 has a transcription-independent regulatory role in NER through MDM2 and/or GADD45. Intriguingly, MDM2, a key regulator in the P53 pathway, can also be affected by oestrogenic signals [42]. A higher repair activity in females may be related to the induction of NER by hormone-driven DNA damage. This is, however, not directly in line with the age effects seen in female DNA repair activity in the present study. Following menopause, there is a significant decrease in oestrogen and progesterone production and thus hormone-driven DNA damage is expected to decrease as well. Nonetheless, considering the potential influence of sex-related hormones on DNA repair, our data indeed suggest the need for considering age when analysing sex differences in DNA repair. We should however interpret these data with caution because the group of females with an age of >70 years (showing the highest NER activity) consists of only seven individuals.

Obesity has been recognized as a risk factor for cardiovascular disease and cancer, and obesity can affect genome stability. Pan *et al.* [43] found a link between functional polymorphisms in some major NER genes and oesophageal cancer risk and reported that this NER-related risk was more evident in overweight/obese individuals and male smokers. Previous studies showed an inverse association between BMI and NER in white blood cells [3,44]. In the current study, we do not observe an overall effect of BMI on NER activity; however, after stratification for sex, the highest NER activity was seen in females with a relatively low BMI (<25 kg/m^2^). This is in line with the findings of a recent breast cancer case-control study, showing that women with a BMI > 25 kg/m^2^ tend to have lower DNA repair capacity levels [16]. Interestingly, a recent mouse study showed that weight loss increased NER activity in various organs [45], suggesting that weight loss may prevent obesity-associated adverse health effects due to the reduction of overall DNA damage. Still, the overall knowledge concerning the impact of body weight/BMI and DNA damage on DNA repair is limited and warrants further research.

Cigarette smoking is considered an established risk factor for cancer. In an earlier study of the hCOMET database, there was no association between smoking and BER activity measured in white blood cells [32]. Likewise, here we report no overall effect of smoking on NER, but some significant differences were found after stratification by sex; male smokers had higher NER activities than non-smoking males. We can speculate that exposure to chemicals in smoke that induce bulky DNA adducts triggers NER activity. However, the number of subjects after stratification is limited and therefore data have to be interpreted with care. Still, the absence of induction of NER in female smokers can partly explain why bulky DNA adduct levels were higher in females than in males when adjusted for smoking dose [46]. This aspect deserves further attention, again taking sex-differences into account.

Overall, our study has demonstrated that host characteristics are in part responsible for the inter-individual variation in NER activity as measured in peripheral blood cells. Among the host factors that were examined, the strongest and most consistent effects were observed for sex and age, which deserve further attention to elucidate the underlying mechanisms. Our results suggest that NER measured by the comet-based *in vitro* DNA repair assay can be considered as a useful endpoint in future biomonitoring studies or studies in clinical settings.

## Data Availability

All data analysed during this study are included in this published article.
